# In Situ Chemical Modification with Zwitterionic Copolymers of Nanofiltration Membranes: Cure for the Trade-Off between Filtration and Antifouling Performance

**DOI:** 10.1021/acsami.2c05311

**Published:** 2022-06-16

**Authors:** Xinyu Zhang, Jiayu Tian, Ruiyang Xu, Xiaoxiang Cheng, Xuewu Zhu, Ching Yoong Loh, Kaifang Fu, Ruidong Zhang, Daoji Wu, Huixue Ren, Ming Xie

**Affiliations:** † School of Civil and Environmental Engineering, Shandong Jianzhu University, Jinan 250101, PR China; ‡ School of Civil Engineering and Transportation, Hebei University of Technology, Tianjin 300401, PR China; § International Education School, Shandong Polytechnic College (SDPC), Jining 272100, PR China; ∥ Department of Chemical Engineering, University of Bath, Bath BA27AY, U.K.

**Keywords:** nanofiltration membrane, zwitterionic copolymer, in situ surface modification, filtration performance, antifouling properties

## Abstract

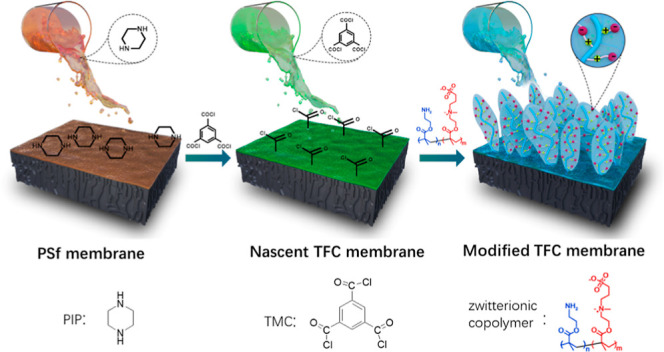

Breaking the trade-off between filtration performance and antifouling property is critical to enabling a thin-film nanocomposite (TFC) nanofiltration (NF) membrane for a wide range of feed streams. We proposed a novel design route for TFC NF membranes by grafting well-defined zwitterionic copolymers of [2-(methacryloyloxy)ethyl]dimethyl-(3-sulfopropyl)ammonium hydroxide (SBMA) and 2-aminoethyl methacrylate hydrochloride (AEMA) on the polyamide surfaces via an in situ surface chemical modification process. The successful grafting of a zwitterionic copolymer imparted the modified NF membranes with better surface hydrophilicity, a larger actual surface area (i.e., nodular structures), and a thinner polyamide layer. As a result, the water permeability of the modified membrane (i.e., TFC-10) was triple that of the pristine TFC membrane while maintaining high Na_2_SO_4_ rejection. We further demonstrated that the TFC-10 membrane possessed exceptional antifouling properties in both static adsorption tests and three cycles of dynamic protein and humic acid fouling tests. To recap, this work provides valuable insights and strategies for the fabrication of TFC NF membranes with simultaneously enhanced filtration performance and antifouling property.

## 1 Introduction

A membrane-based water treatment process is one of the key technologies to solve the problems of water shortage and drinking water safety.^1^ Nanofiltration (NF) plays a pivotal role in the reuse of wastewater and desalination of brackish water, which not only produces high-quality drinking water at a lower cost compared to reverse osmosis (RO) but also separates the small molecules more effectively than ultrafiltration.^2,3^ As the state-of-the-art membrane material for NF technology, thin-film composite (TFC) membranes dominate the current NF industrial market. However, there are still hindrances to the application of TFC NF membranes, such as relatively low permeability and severe membrane fouling.^3−6^ Therefore, improving antifouling performances and perm-selectivity of membranes for NF filtration is of paramount importance to push the boundary outward for a wider NF application.

It is established that the interaction between the membrane surface and foulants dominates the initial stage of membrane fouling.^6,7^ Consequently, there is extensive research interest in the antifouling modification for enhancing TFC membrane surface hydrophilicity via grafting of hydrophilic materials, which could facilitate the establishment of a hydrate layer near the membrane surfaces to mitigate the undesirable adhesion of foulants.^8^ Among them, poly(ethylene glycol) (PEG) has been a widely used hydrophilic material on account of its neutral charge and water bonding ability via hydrogen bonding.^9^ However, PEG is easy to be oxidized and cannot maintain its hydrophilicity in long-term filtration^10^ On the other hand, surface hydrophilicity is also improved via functionalization with hydrophilic nanomaterials,^11−13^ such as silica nanoparticles, carbon nanotubes, and graphene oxides. However, it is difficult for these nanomaterials to be distributed evenly on the membrane surface or within the membrane matrix because of the agglomeration of these nanoparticles.^12,13^ Vulnerability to loss is another important issue because of the lack of effective chemical bonding between nanomaterials and the membrane surface.^12,13^

Zwitterion-based polymers that possess both positively and negatively charged functional groups have triggered widespread interest as an excellent antifouling material due to their overall charge neutrality and high hydrophilicity.^14−18^ Hence, there are many post-modification methods for commercial polyamide TFC membranes with zwitterionic polymers, for example, electrostatic coating, redox reactions, initiated chemical vapor deposition, UV-initiated radical grafting, and surface-initiated atom transfer radical polymerization (SI-ATRP).^16−21^ Especially for the SI-ATRP process, excellent fouling resistance can be achieved due to the controlled architecture of zwitterionic polymer brush layers. Considering that the zwitterionic polymer brush layer can only delay the appearance of membrane fouling as much as possible, it is not inevitable to apply the membrane cleaning process to restore membrane performance. However, the polydopamine transition coating that was used to introduce the SI-ATRP initiator exhibited poor acid and alkali resistance, and thus, the stability of the resultant-modified layer was of paramount interest for practical deployment in the membrane cleaning process.^62−64^ Many studies on surface grafting modification of antifouling polyamide TFC membranes have also pointed out the phenomenon that an additional modification layer induced by hydrophilic polymers can significantly increase the permeability resistance.^8,22^ In other words, there is indeed a trade-off between the improved performance and the reduced fouling tendency induced by the surface modification of polyamide TFC membranes. Therefore, simultaneous enhancement of membrane flux is another key issue that cannot be avoided in the process of exploring antifouling TFC membranes.

In situ surface chemical modification is a facile and scalable technique in which the hydrophilic materials are in situ robustly grafted onto the nascent polyamide layer by reaction with surface acyl chloride groups in the process of membrane preparation. Hu et al.^23^ fabricated a polyamide RO membrane by in situ surface grafting of small-molecule zwitterions to enhance water permeability and fouling-resistance properties. An et al.^24^ reported surface zwitterionic polyamide NF membranes, and Chiao et al.^25^ fabricated a polyamide forward osmosis membrane with *N*-aminoethyl piperazine propane sulfonate (small-molecule zwitterion) through the in situ surface chemical modification technique. Enhanced antifouling properties and filtration performance were reported for the modified membranes. Therefore, the in situ surface chemical modification approach is another promising method for chemically bonding zwitterionic polymers to the membrane surface to break the trade-off between antifouling property and filtration performance. However, most zwitterionic candidates with desirable anchor groups are small molecules with limited options.^26^

**Figure 1:**
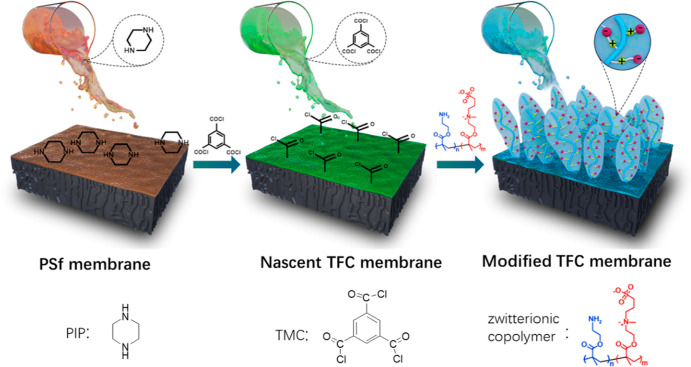
Schematic diagram of the in situ surface chemical modification on the polyamide surface using zwitterionic copolymer brushes.

In this study, a novel zwitterionic copolymer was synthesized by polymerization of 2-aminoethyl methacrylate (AEMA) and [2-(methacryloyloxy)ethyl]dimethyl-(3-sulfopropyl)ammonium hydroxide (SBMA) and employed as an effective grafting material for synergistically enhancing filtration and antifouling properties of TFC NF membranes. As illustrated in Figure 1, zwitterionic poly(SBMA) segments act as antifouling polymer brushes, while the primary amine groups of poly(AEMA) segments perform as anchors to chemically bind with the acyl chloride groups dangling on the nascent polyamide surface through the in situ surface chemical modification technique. The modified NF membranes are first systematically characterized to confirm the polyzwitterionic grafting. We then evaluated the effect of the polyzwitterionic grafting concentration on the morphologies of the resulting polyamide layers. Lastly, we examined the filtration and antifouling properties of the modified NF membranes. Our fabrication method and results shed light on the preparation of zwitterionic material-functionalized polyamide TFC membranes to break the trade-off between antifouling properties and filtration preformation.

## 2 Materials and Methods

### 2.1 Materials and Chemicals

#### 2.1.1 Materials and Chemicals

2-Aminoethyl methacrylate (AEMA, 98%, Macklin Inc) and [2-(methacryloyloxy)ethyl]dimethyl-(3-sulfopropyl)ammonium hydroxide (SBMA, 99%, J&K Scientific) were employed as monomers to synthesize the zwitterionic copolymer P[SBMA-*co*-AEMA] as reported by Han et al.,^14^ and the detailed process was illustrated in the Supporting Information (S1). The *M*_n_ and *M*_w_ of the resultant zwitterionic copolymer were 3216 and 4237 Da, respectively, which were measured by the gel permeation chromatography experiments at room temperature with DI water as the mobile phase and polymethyl methacrylate as the standard. 1,3,5-Benzenetricarbonyl trichloride (TMC, 98%, J&K Scientific), piperazine (PIP, >99%, J&K Scientific), and polyethylene glycols (PEG, 99%) with different molecular weights between 200 and 800 g/mol were purchased from Sigma-Aldrich (China). 2,2′-Azoisobutyronitrile (AIBN, 99%), dimethyl sulfoxide (DMSO, 99.5%), *n*-hexane (99.5%), sodium hydroxide (NaOH, 99.9%), sodium chloride (NaCl, 99.9%), phosphate-buffered saline (PBS, pH 7.4), sodium sulfate (Na_2_SO_4_, 99.9%), bovine serum albumin (BSA, 98%, molecular weight 68 kDa), and humic acid (HA, >90%) were obtained from J&K Scientific Ltd. (China). Except for the TMC organic solution, all solution preparations used Milli-Q water as the solvent.

#### 2.1.2 NF Membrane Fabrication via In Situ Surface Chemical Modification

As illustrated in Figure 1, the modified TFC NF membranes were prepared via the in situ surface chemical modification. The PES support membrane was soaked in the 1.0 wt % PIP solution containing 0.5 wt % NaOH in water for 60 s, and excess PIP solution was swept from the membrane surface using an air knife. Then, the resultant PIP-adsorbed PES support was coated with the 0.1 wt % TMC/*n*-hexane solution. After a 30 s reaction, the nascent polyamide NF membrane was obtained and labeled as the pristine TFC membrane after being heat-cured in a 60 °C water bath for 5 min. In the case of surface grafting of the P[SBMA-*co*-AEMA] copolymer, the in situ surface chemical modification process of preparing the modified NF membranes was similar to that of the pristine TFC membrane prior to the heat-curing step, which was carried out by pouring P[SBMA-*co*-AEMA] aqueous solution with different concentrations (0.5, 1.0, and 1.5 wt %) on the nascent polyamide surface. The reaction time between the amine groups of P[SBMA-*co*-AEMA] and the acyl chloride group on the polyamide surface was set for 2 min to form a polyzwitterionic graft. The modified TFC membrane was designated as TFC-5, TFC-10, and TFC-15, corresponding to the zwitterionic copolymer concentration, respectively.

#### 2.1.3 Membrane Characterization

Attenuated total reflectance Fourier transform infrared spectroscopy (ATR–FTIR, Nexus 470, Nicolet) and X-ray photoelectron spectrometry (XPS, ESCALAB 250Xi, ThermoFisher) were employed to detect the functional groups and elemental components of the membrane surfaces, respectively. Surface and cross-sectional topographies of NF membranes were both characterized by field-emission scanning electron microscopy (SU7000, Hitachi). Atomic force microscopy (AFM, Dimension Edge, Bruker) was used to quantify the surface roughness with a scanning area of 5 μm × 5 μm in a tapping mode, and the detailed method can be found in the Supporting Information. Surface zeta potential was determined using a ζ-potential analyzer (SurPASS 3, Anton Paar) with a pH range from 3 to 9. The rejection experiment of neutral organic solutes (200 ppm) with different molecular weights (200, 300, 400, 600, and 800 Da) was carried out to determine the effective molecular weight cut-off (MWCO) of the resultant NF membranes.

Surface hydrophilicity was assessed by analyzing the water contact angle (CA) by employing a dynamic CA goniometer (OCA20, Dataphysics). In addition, the water CA change was also determined to evaluate the stability of zwitterionic copolymer brushes on the polyamide surface before and after chemical or physical stress.^27^ Particularly, chemical stress was caused by contacting the polyamide surfaces with HCl solution (pH = 3), NaOH solution (pH = 10) or 0.6 M NaCl solution for 60 min, followed by thorough washing with DI water. Physical stress is applied by soaking the membrane in a sonication water bath for 30 min.

#### 2.1.4 Performance Evaluations of the NF Membranes

Water permeance (*A*, L·m^–2^·h^–1^·bar^–1^) and salt rejections (*R*_s_, %) were determined by using a cross-flow setup with the effective filtration area of 16.8 cm^2^ to assess the filtration performance of NF membranes. The system pressure, cross-flow velocity, and temperature of the feed stream were maintained at 5 bar, 8.5 cm/s, and 25 °C, respectively. The single salt concentration of feed solution was 1000 ppm. The water permeance (*A*, L·m^–2^·h^–1^·bar^–1^) and rejection property (*R*_*s*_, %) were calculated using the following eqs 1 and 2, respectively:
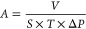
1
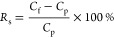
2where Δ*P* and *S* are denoted as the *trans*-membrane pressure (bar) and the membrane filtration area (m^2^), respectively; *V* (L) is the permeate volume over a period of interval *T* (h); *C*_f_ and *C*_p_ represent the salt concentrations of the feed and permeate solutions, respectively.

#### 2.1.5 Static Adsorption Test

The static adsorption tests of the modified membranes were performed by immersing the membrane coupon (10 cm^2^) into a 20 mL BSA solution (2000 ppm), where the pH was adjusted to 7.4 by using 0.1 M PBS.^28^ The unmodified TFC NF membrane was employed as a control. After achieving adsorption equilibrium (about 24 h), the membrane coupon was taken out and the residual BSA concentration in solution was determined by using a UV–vis spectrophotometer (UV-2600, Shimadzu), and correspondingly, the BSA adsorption amount of the membrane coupon was measured using the following eq 3
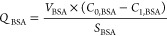
3where *Q*_BSA_ (μg·cm^–2^) and *V*_BSA_ (L) were the BSA adsorption amount and BSA solution volume, respectively; *C*_0,BSA_ (g·L^–1^) and *C*_1,BSA_ (g·L^–1^) were the BSA concentrations in solution before and after the static adsorption test, respectively; and *S*_BSA_ (cm^2^) was the membrane area.

#### 2.1.6 Membrane Antifouling Performance

The dynamic membrane fouling test was also conducted at a cross-flow velocity of 8.5 cm/s for the feed solution, which was composed of organic foulants (500 ppm) and inorganic composites (16 mM NaCl, 1 mM NaHCO_3_, and 1 mM CaCl_2_).^18^ Herein, BSA and HA were employed as the model organic foulants of protein and natural organic matter (NOM), respectively. First, the fouling experiment was stabilized with the inorganic solutions at 25 °C, and the initial water flux was fixed at 18 L·m^–2^·h^–1^ (denoted as *J*_0_) by adjusting the operating pressure. Next, aliquots of BSA/HA solutions were charged into the feed solution to start the fouling experiment. The fouling experiment was performed for 3 h, and the final flux was designated as *J*_t_. Subsequently, foulant solution was replaced with fresh water to wash the fouled membrane for 30 min. The recovered water flux (denoted as *J*_r_) was characterized by using inorganic solution as the feed solution. Then, the next cycle fouling experiment was started after adding an organic foulant into the inorganic solution. Each of the membranes was assessed with one or three cycles. The flux decline ratio (FDR) and flux recovery ratio (FRR) were calculated according to the following eqs 4 and 5, respectively
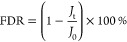
4
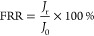
5

## 3 Results and Discussion

### 3.1 Membrane Characterizations

**Figure 2:**
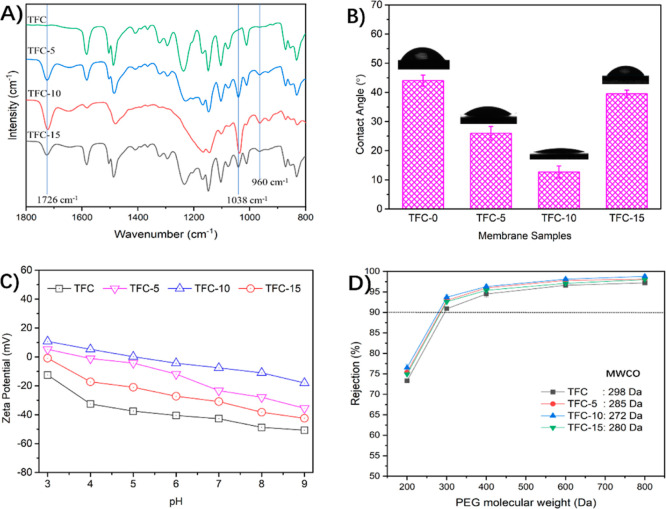
(A) ATR–FTIR spectra, (B) water CA, (C) zeta potential, and (D) variation of PEG rejection ratios with different molecular weights of the pristine TFC, TFC-5, TFC-10, and TFC-15 membranes. The error bar represents the standard deviation from triple measurements.

The chemical structures of the pristine and modified TFC NF membranes were characterized by ATR–FTIR and XPS. Figure 2A illustrates the characteristic functional groups in the membrane surfaces in the 1800–800 cm^–1^ wavenumber region of the FTIR spectra. After the modification with the zwitterionic copolymers, three new peaks at 1726, 1038, and 960 cm^–1^ are observed for all three modified membranes compared to the pristine TFC membrane, which can be ascribed to the carbonyl in the ester group, the symmetric stretch of the sulfonate group, and the quaternary amine headgroups belonging to the zwitterionic copolymers, respectively.^29^ This observation demonstrates that the zwitterionic copolymer brushes have been chemically grafted to the TFC membrane surface via the in situ surface chemical modification approach.

The existence of the zwitterionic copolymer on the membrane surfaces is also confirmed by the XPS results. Based on the chemical structure, the zwitterionic copolymer could enrich the polyamide surface with more oxygen atoms. Therefore, the surface N/O ratio can reflect the grafting degree of the zwitterionic copolymer, which means that a higher grafting degree of the zwitterionic copolymer could result in a lower surface N/O ratio. As can be seen clearly from Table 1, the surface N/O ratio is significantly reduced after the grafting of zwitterionic copolymers with different concentrations, for example, 0.5, 1.0, and 1.5 wt %. Among them, the TFC-10 membrane exhibits the lowest surface N/O ratio of 0.34 and thus gains the highest grafting degree. However, compared to the TFC-10 membrane, a lower grafting degree of the TFC-15 membrane is observed as demonstrated by the higher surface N/O ratio of 0.63. This observation is mainly driven by the fact that a higher concentration could restrict the chain mobility of the zwitterionic copolymer in solution and thus reduce the grafting ratio.^30,31^ Particularly, as the characteristic element of the zwitterionic copolymer, a sulfuric element is detected in all three modified TFC membranes, especially for the TFC-10 membrane (3.0 mol %), but it is negligibly detected in the pristine TFC membrane (0.2 mol %). Taken together, the above results confirm the successful grafting of zwitterionic copolymer brushes onto the polyamide surface.

**Table 1:** Elemental Compositions of the Pristine and Modified NF Membranes Measured by XPS

	atomic percent (mol %)				atomic ratio		
	C	N	O	S	N/C	O/C	N/O
zwitterionic copolymer^a^	51.1	7.4	34.8	6.7	0.14	0.68	0.21
TFC membrane	77.7	13.4	14.2	0.2	0.17	0.18	0.94
TFC-5 membrane	68.7	7.4	21.3	2.6	0.11	0.31	0.35
TFC-10 membrane	68.9	7.2	21.0	3.0	0.10	0.30	0.34
TFC-15 membrane	73.6	9.9	15.7	0.8	0.13	0.21	0.63

a Elemental composition of the zwitterionic copolymer was employed as a reference and calculated based on its chemical structure.

The surface wettability of the pristine and modified NF membranes was evaluated by measuring the water CA (Figure 2B). The pristine TFC membrane presents the highest water CA of 44°. After modification by the zwitterionic copolymer with concentrations of 0.5, 1.0, and 1.5 wt %, the water CA of three modified membranes remarkably reduces to 26, 12.7, and 39.5°, respectively. As a result, the TFC-10 membrane exhibits the lowest CA and may possess the optimum antifouling property. The trend of decreasing the water CA (i.e., increasing surface hydrophilicity) is consistent with that of the surface N/O ratio (Table 1), indicating that enhanced surface hydrophilicity can be ascribed to the introduction of a zwitterionic copolymer onto the membrane surface.

CA measurement was also employed to estimate the stability of the modified layer. The water CA of the TFC-10 membrane was re-measured after the surface was challenged by chemical or physical stresses, such as a pH 3 solution (HCl), a pH 10 solution (NaOH), a 0.6 M NaCl solution, or a sonicating water bath. Compared to the membrane detected immediately after modification, the water CAs have negligible changes as illustrated in Figure S2 (Supporting Information), demonstrating that the zwitterionic copolymer is chemically and irreversibly grafted to the membrane surface. FT-IR spectrum analysis and membrane performance are also carried out in the stress protocol. Key membrane physicochemical properties have no significant change compared to those that were subjected to DI water (Figures S3 and S4 in the Supporting Information), which could warrant long-term stability of antifouling properties. Indeed, the in situ chemical modification method provides better resistance against the chemical and physical stress than the SI-ATRP method using the polydopamine transition coating, especially in the membrane cleaning process.

The surface charge of the resultant membranes was characterized by the streaming potential measurements, and the corresponding results are illustrated in Figure 2C. As the polyamide layer of the TFC NF membrane is polymerized by the reaction between PIP and TMC, surface charge behavior is dominated by the unreacted carboxylic and amine groups.^32^ As the pH increases from 3 to 9, the zeta potential gradually becomes more negative for the pristine TFC membrane due to the deprotonation of carboxyl groups. On the other hand, after grafting the zwitterionic copolymer to the membrane surface, the zeta potential curves are shifted upward because of the shielding effect of the zwitterionic copolymer on carboxyl groups dangling on the polyamide surface, which is consistent with the previous results.^16,17,33^ The TFC-10 membrane exhibits the nearest electrically neutral surface. In addition, the trend of zeta potential curves also agrees well with that of the surface N/O ratio.

The MWCOs of these NF membranes were calculated on the basis of 90% rejection of a series of neutral PEG solutions with different molecular weights. Compared to the MWCO value of the TFC membrane (298 Da) and the TFC-15 membrane (280 Da) as presented in Figure 2D, the zwitterionic copolymer-modified TFC membrane exhibits a lower MWCO value (i.e., 285 and 272 Da for TFC-5 and TFC-10 membranes, respectively). This result may be driven by the overlay of the zwitterionic copolymer brushes on the polyamide surface.^18^ NF membranes with a smaller pore size could facilitate the enhancement of the separation performance.

### 3.2 Membrane Morphology

**Figure 3:**
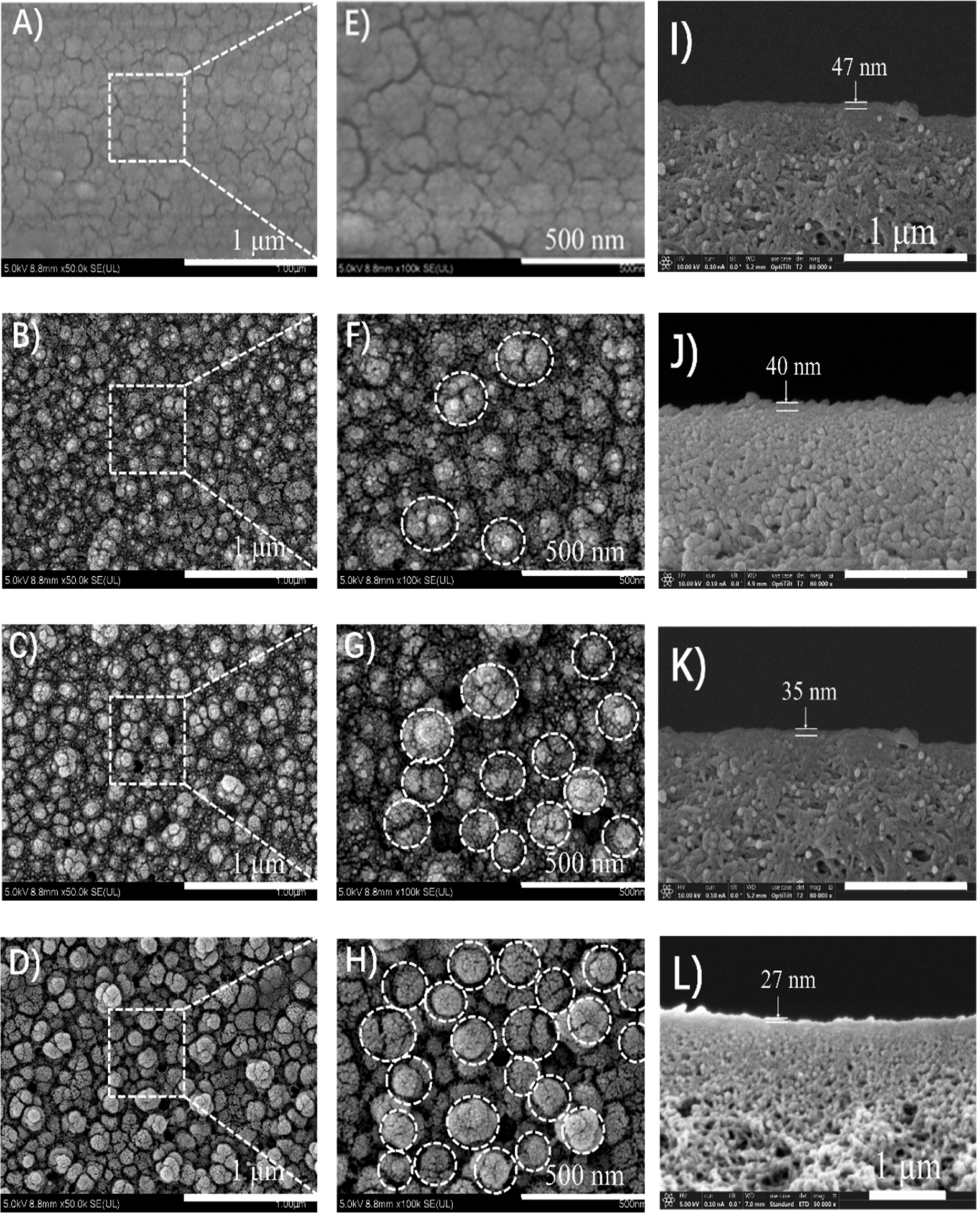
Surface SEM images of the polyamide active layer of (A,E) pristine TFC, (B,F) TFC-5, (C,G) TFC-10, (D,H) TFC-15 membrane; and the cross-section images of (I) pristine TFC, (J) TFC-5, (K) TFC-10, and (L) TFC-15 membranes.

Surface morphology of NF membranes plays a crucial role in separation and antifouling properties.^34,35^ The SEM micrographs of NF membranes modified by the zwitterionic copolymer with different grafting concentrations are determined and illustrated in Figure 3. The pristine TFC membrane exhibits the smoothest surface with small, nodular structures (Figure 3A,E), which are formed by a rapid reaction between PIP and TMC. Surprisingly, as observed in Figure 3B–D,F–H, these nodules gradually become more obvious and homogenous on the modified membrane surfaces as the grafting concentration of the zwitterionic copolymer increases from 0.5 to 1.5 wt % at the same time. Simultaneously, the size of these nodules was not changed significantly. It is noteworthy that the surface morphology of the polyamide layer is mainly dominated by the initial location on the supports and the subsequent diffusion rate of PIP monomers into the organic solution.^34,36^ Considering that all the polyamide layers are prepared through the same reaction between TMC and PIP on the same kind of PES support in our fabrication protocol, it is hypothesized that the grafting concentration of the zwitterionic copolymer has affected the interfacial polymerization process and thus results in different surface morphologies of modified NF membranes.

**Figure 4:**
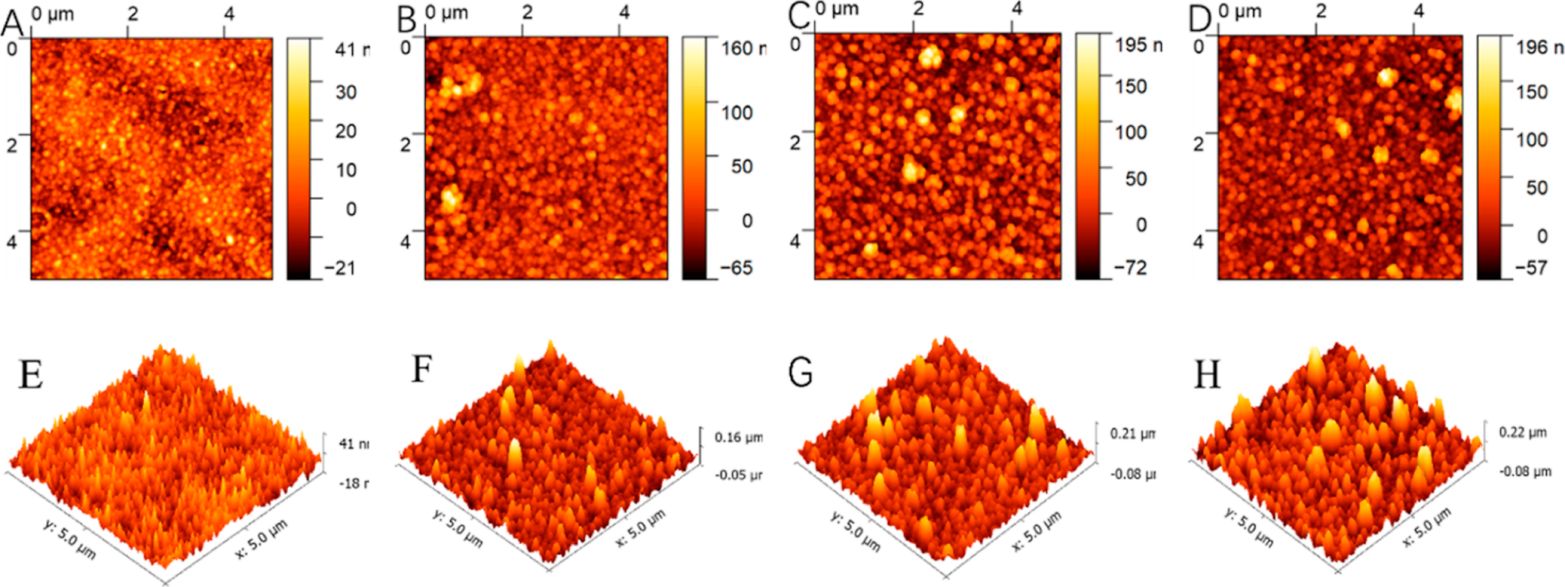
AFM 3D images of (A,E) pristine TFC, (B,F) TFC-5, (C,G) TFC-10, and (f) TFC-15 membranes.

The AFM images of the pristine and modified TFC membranes are also displayed in Figure 4. Compared to Figures 3 and 4, the AFM results further verify the nodular structures in the SEM images. As summarized in Table 2, the pristine TFC membrane has an average roughness (*R*_a_) of 5 ± 0.6 nm, a root-mean-square roughness (*R*_rms_) of 7.2 ± 0.7 nm, a maximum roughness (*R*_max_) of 41.7 ± 6.2 nm, and an SAD of 1.7 ± 0.2%. Compared to the pristine TFC membrane, surface roughness for the modified TFC membranes increases remarkably, which is beneficial for enhancing the filtration performance.^34^ In particular, the TFC-15 membrane exhibits the highest roughness (*R*_rms_ = 40.8 ± 3.9 nm, *R*_a_ = 31.4 ± 1.6 nm, *R*_max_ = 243.3 ± 47.6 nm, and SAD = 26.3 ± 0.3%). Meanwhile, it is also noteworthy that this is followed by the TFC-10 membrane (*R*_rms_ = 34.5 ± 5.7 nm, *R*_a_ = 21.9 ± 3.7 nm, *R*_max_ = 231.8 ± 44.7 nm, and SAD = 21.2 ± 0.7%) and the TFC-5 membrane (*R*_rms_ = 23.5 ± 1.7 nm, *R*_a_ = 16.7 ± 0.8 nm, *R*_max_ = 174.7 ± 57.6 nm, and SAD = 12.8 ± 0.8%). The difference in surface roughness of these TFC membranes could be further attributed to the grafting concentration of the zwitterionic copolymer employed in the in situ surface chemical modification process.

**Table 2:** Surface Roughness Determined by AFM for Pristine TFC, TFC-5, TFC-10, and TFC-15 Membranes

membranes	TFC	TFC-5	TFC-10	TFC-15
*R*_rms_^a^ (nm)	7.2 ± 0.7	23.5 ± 1.7	34.5 ± 5.7	40.8 ± 3.9
*R*_a_^b^ (nm)	5 ± 0.6	16.7 ± 0.8	21.9 ± 3.7	31.4 ± 1.6
*R*_max_^c^ (nm)	41.7 ± 6.2	174.7 ± 57.6	231.8 ± 44.7	243.3 ± 47.6
SAD^d^ (%)	1.7 ± 0.2	12.8 ± 0.8	21.2 ± 0.7	26.3 ± 0.3

a Root mean squared roughness (*R*_rms_): the RMS deviation of the peaks and valleys from the mean plane.

b Average roughness (*R*_a_): arithmetic average of the absolute values of the surface height deviations measured from the mean plane.

c Maximum roughness (*R*_max_): the maximum vertical distance between the highest and lowest data points in the image following the plane fit.

d Surface area difference (SAD): the increase in surface area (due to roughness) over a perfectly flat plane with the same projected area.

**Figure 5:**
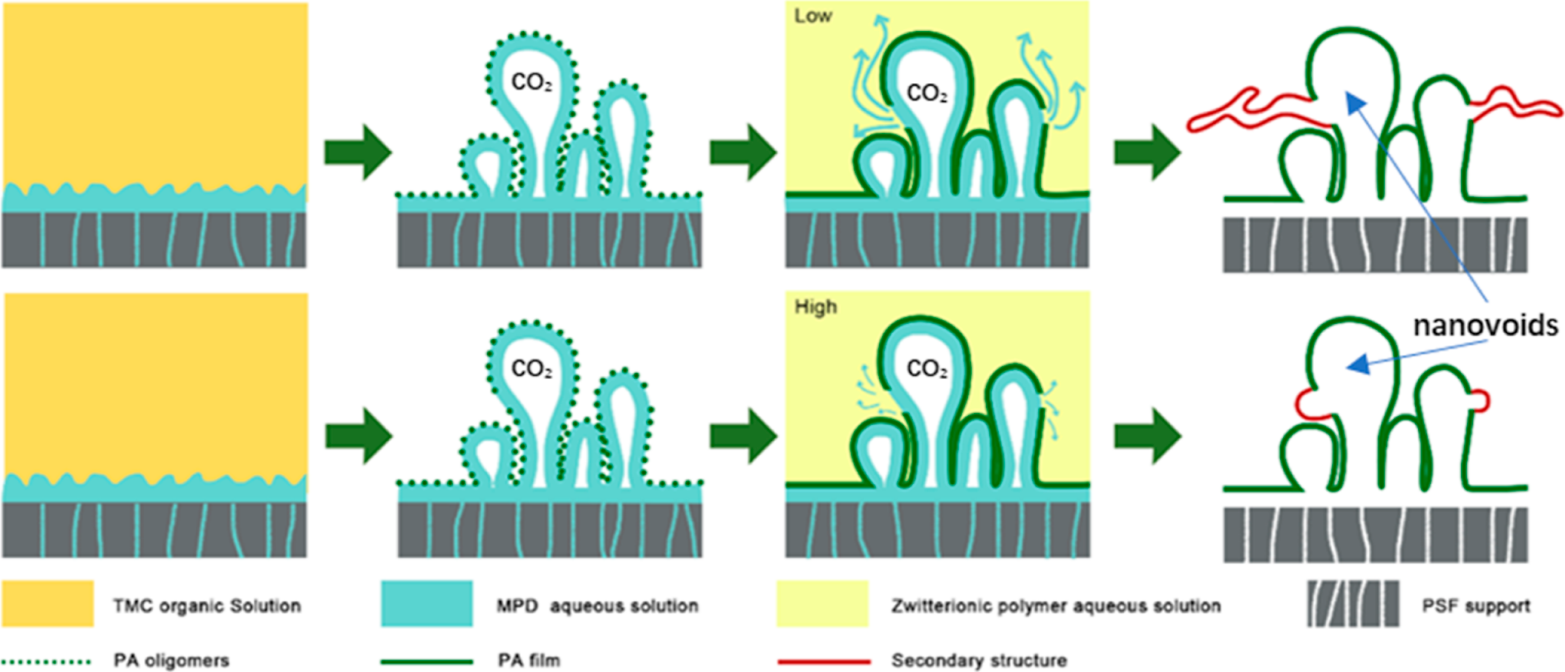
Formation mechanisms of the nodular layer (represented by green solid lines) and the exterior layer (represented by red dotted lines).

Furthermore, it can be found that the nodular structures on the membrane surface (Figure 3H–E) are gradually diminished by the exterior layer when the grafting concentration of the zwitterionic copolymer decreases from 1.5 to 1.0, 0.5, and 0 wt %. As a result, it concludes that the polyamide layer of the pristine TFC membrane possesses a dual-layer structure with a nodular layer and an additional exterior layer. We attributed this phenomenon to CO_2_ degassing during interfacial polymerization.^37−40^ However, when the zwitterionic copolymer solution is added in the conventional IP process, the additional external layer becomes less and less visible around nodular structures. This phenomenon can be explained by the fact that the CO_2_ degassing process and the formation of exterior layer are both deeply suppressed by zwitterionic copolymer solution. Increased viscosity and surface tension of zwitterionic copolymer solution are disadvantageous to the CO_2_ degassing process and the formation of the exterior layer. It is noteworthy that the viscosity and surface tension of zwitterionic copolymer solutions increase with their concentrations (Figure S5 in the Supporting Information). Indeed, this hypothesis can be further confirmed by the change in thickness of polyamide layers. As seen in Figure 3I–L, the thicknesses of polyamide layers are gradually decreased from 47 nm for the pristine TFC membrane to 40 nm for TFC-5, 35 nm for TFC-10, and 27 nm for the TFC-15 membrane, demonstrating the effective inhibition of the formation of the exterior layer by the zwitterionic copolymer solutions (Figure 5).

### 3.3 Membrane Separation Performance

**Figure 6:**
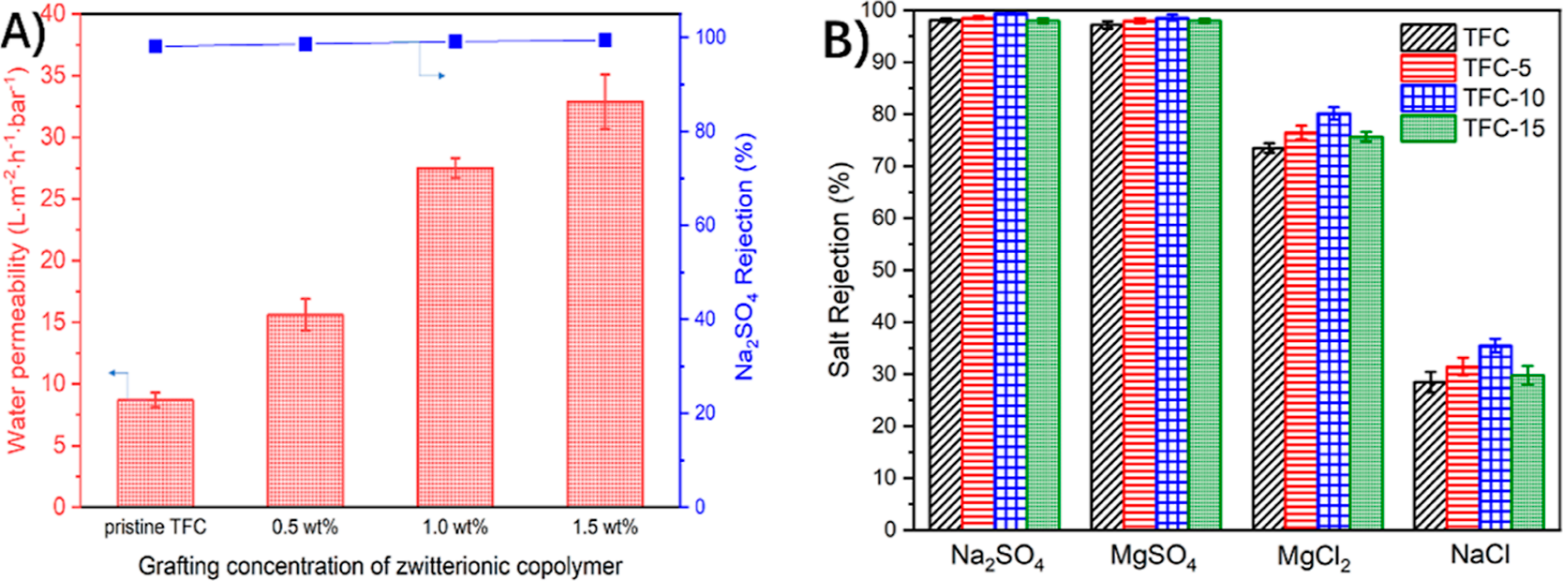
(A) Water permeability and Na_2_SO_4_ rejection of pristine and modified NF membranes fabricated using different zwitterionic copolymer grafting concentrations, (B) Salt rejection of the pristine TFC, TFC-5, TFC-10, and TFC-15 membranes.

We have demonstrated that the grafting concentration of zwitterionic copolymers has an important influence on the membrane interface characteristics. To further correlate the unique membrane interfacial characteristics to membrane separation performance, the resultant NF membranes are evaluated to examine water permeability and salt separation performance. As seen in Figure 6A, the pristine TFC membrane has a water permeability of 8.7 L·m^–2^·h^–1^·bar^–1^. Specifically, accompanied by the concentration increase of the zwitterionic copolymer solution from 0.5 to 1.0 and 1.5 wt %, the water permeability is remarkably enhanced from 15.6 to 27.5 and 32.9 L·m^–2^·h^–1^·bar^–1^, which is correspondingly 1.7, 3.1, and 3.7 times higher than that of the pristine NF membrane, respectively. As for Na_2_SO_4_ rejection, the salt rejection is 98.1, 98.6, 99.1, and 99.4% for TFC, TFC-5, TFC-10, and TFC-15 membranes, respectively, indicating that the polyamide layer is well prepared after grafting the zwitterionic copolymer.

The in situ surface chemical modification process using zwitterionic copolymers not only results in abundantly nodular structures with higher roughness that increases the permeable area of water molecules but also reduces the polyamide layer thickness that substantially shortens the passage path of water molecules.^28,41−43^ In addition, the zwitterionic copolymer highlights strong water-bonding capability via electrostatic interaction, which binds water molecules toward the polyamide surface.^44^ Taken together, the higher water permeability of the resultant NF membranes is responsible for the thinner polyamide layer, larger surface area, and more hydrophilic interface.

Separation performance of the pristine and modified NF membranes is accessed by employing four types of salts, that is, Na_2_SO_4_, MgSO_4_, MgCl_2_, and NaCl. As illustrated in Figure 6B, the rejection of all membranes for divalent salts is higher than that for monovalent salts, which follows the order of Na_2_SO_4_ ≈ MgSO_4_ > MgCl_2_ > NaCl, which was consistent with results in previous literature.^18,28^ It is well known that the synergistic effect of spatial repulsion and Donnan repulsion dominates the rejection of the divalent salt. In our modification approach (e.g., the TFC-10 membrane), the zwitterionic copolymer could shield the carboxylic groups on the polyamide surface, weakening the Donnan repulsion effect.^45^ However, Na_2_SO_4_ and MgSO_4_ rejections of the modified membranes (e.g., the TFC-10 membrane) are still maintained at levels as high as those of the pristine TFC membrane. This is because the narrowed pore size of the modified membrane could strengthen the size repulsion effect. Therefore, size repulsion could be considered as the main factor in divalent salt rejection. The data of monovalent salt rejection are also illustrated in Figure 6B, and the pristine TFC membrane displays 29% rejection of NaCl. Compared to this, NaCl rejection of the TFC-10 membrane is significantly increased by 25%. This may also be due to the decrease in membrane pore size, which is conducive to promoting NaCl rejection through spatial repulsion. Taken together, excellent separation performance of the zwitterionic copolymer-modified NF membrane can be successfully achieved by the in situ surface chemical modification method.

### 3.4 Anti-adhesion Properties of TFC Membranes

**Figure 7:**
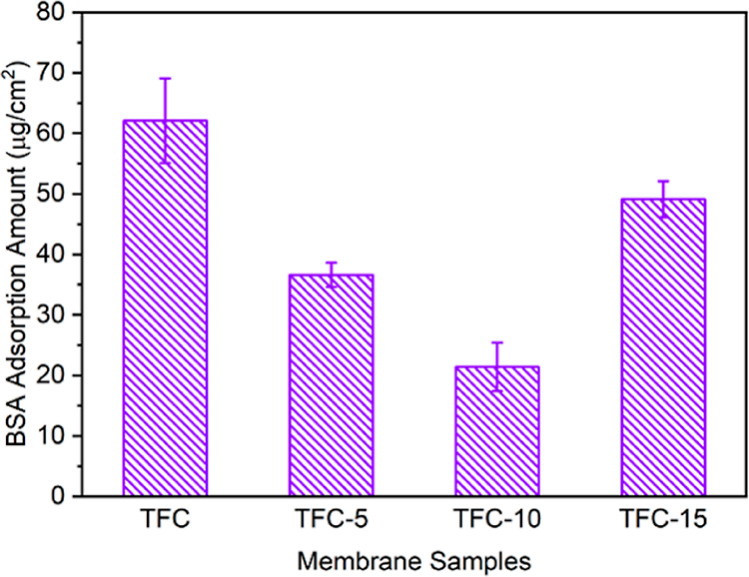
Data of static adsorption tests using BSA as the model foulant. Error bars represent the standard deviation from triple measurements obtained from two membrane coupons.

Static BSA adsorption tests were performed to appraise the antifouling performance of the resultant NF membranes. Figure 7 plotted the data of the static BSA adhesion test. Although the pristine TFC membrane has the smoothest surface, its BSA adsorption capacity is the largest (62 μg·cm^–2^). By contrast, a significant reduction in the BSA adsorption capacity is observed after the grafting of the zwitterionic copolymer. Despite having a relatively high surface roughness, the TFC-10 membrane displays the best antifouling performance with 66% reduction in BSA adsorption compared to that of the pristine TFC membrane.

Previous studies have reported that the surface roughness and surface wettability of the polyamide layer have an important influence on the fouling tendency of the polyamide surface.^35,40^ As discussed in Section 3.2, the surface roughness of modified NF membranes is obviously higher than that of the pristine TFC membrane and enhances with the increase in zwitterionic copolymer grafting concentration. Nevertheless, the BSA adsorption amount shows the opposite trend but is consistent with that of the water CA for the modified TFC membranes (Figure 2B). Besides, the CA value of the TFC-10 membrane decreases by 71% compared with that of the pristine TFC membrane, which is much higher than the SAD value, which increases by 21%. Consequently, it is hypothesized that surface wettability is the dominating factor for the fouling tendency of NF membranes fabricated in this work.

### 3.5 Antifouling Performance of TFC Membranes in Dynamic Fouling Tests

**Figure 8:**
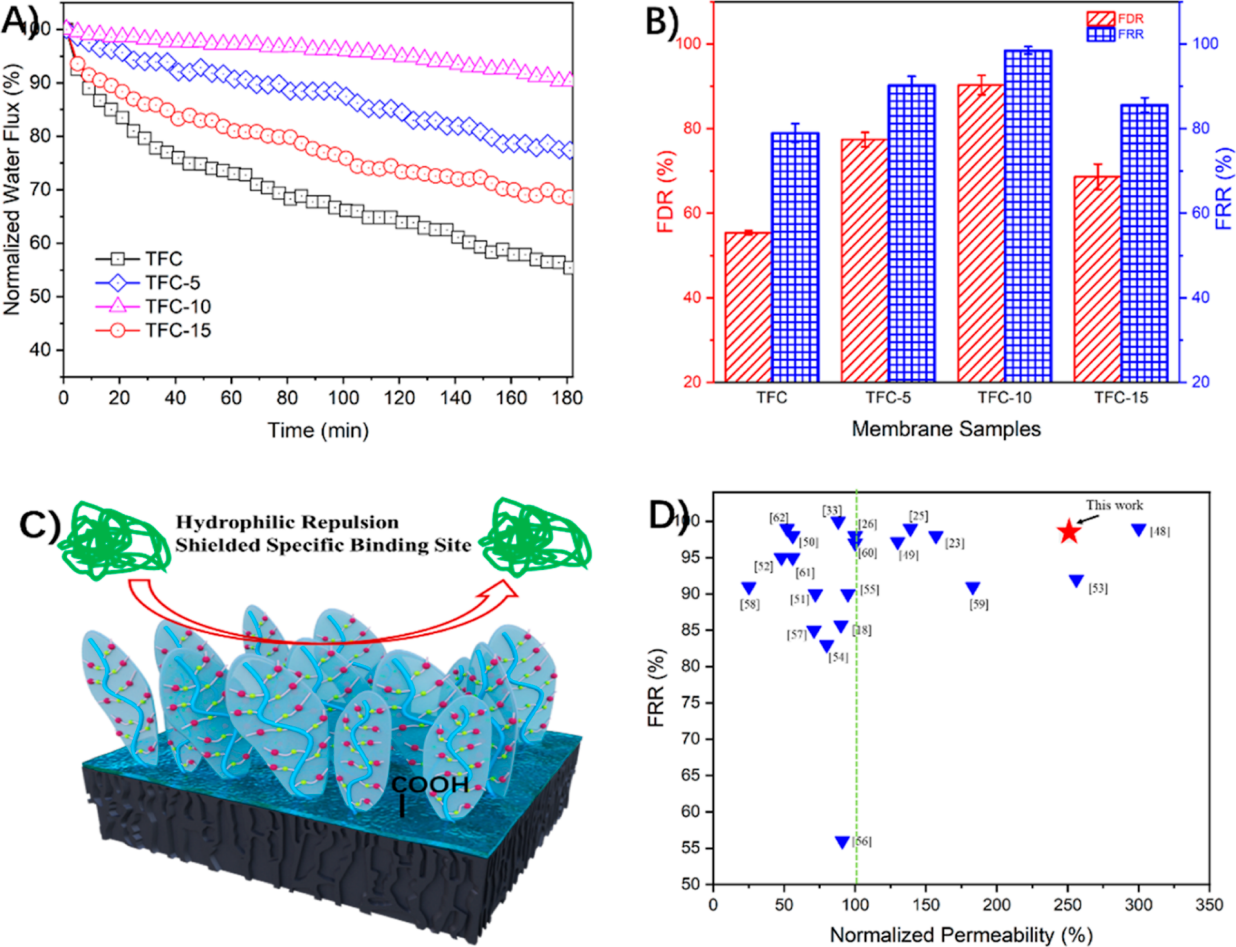
(A) Normalized flux varies due to BSA fouling and (B) corresponding FDR and FRR for the pristine and modified TFC NF membranes. (C) Schematic illustration of the antifouling mechanisms of the modified TFC NF membrane. (D) Summary of membrane performances of the TFC-10 membrane in this work and the polyamide TFC membranes reported in the literature with regard to normalized permeability vs FRR. Fouling conditions were as follows: BSA foulant (500 ppm) and inorganic composites (16 mM NaCl, 1 mM NaHCO_3_, and 1 mM CaCl_2_) were used as the feed solution. The initial water flux was fixed at 18 L m^–2^ h^–1^ by adjusting the operation pressure.

To evaluate the effect of in situ surface chemical modification on the correlation between membrane properties and dynamic fouling tendencies, the normalized water flux as the function of BSA fouling filtration for the fabricated TFC membranes in the dynamic fouling process is plotted in Figure 8A. The FDR values after the fouling stage and the FRR values after the cleaning process are also illustrated in Figure 8B. In particular, membrane fouling potency is effectively alleviated after grafting of zwitterionic copolymer brushes, which is demonstrated by the mild decrease in the flux profile of the TFC-10 membrane. On the other hand, the water flux of the pristine TFC membrane shows a 45% reduction in its original flux and can only be restored to ∼79% (Figure 8B). In contrast, the TFC-10 membrane exhibits the optimum antifouling properties, as displayed with merely less than 10% flux decline and more than 98% recovery flux.

Taken together, the static and dynamic BSA fouling experiments have both supported and verified the effectiveness of the in situ surface chemical modification method to fabricate zwitterionic copolymer-modified NF membranes. The excellent antifouling properties of the zwitterionic copolymers can be ascribed to two key mechanisms, as illustrated in Figure 8C.^16,17,33^ First, zwitterionic copolymers possess a strong hydration capability via ionic solvation with water molecules, which could form an energetic and steric barrier between the membrane surface and foulants. Second, the shielding effect on carboxylate groups by zwitterionic copolymers could play an important role in alleviating membrane fouling induced by the “calcium bridging” effect.

In addition, the normalized permeability and antifouling property of the TFC-10 membrane in this work are compared with those reported in the literature, as illustrated by the values of the relevant indicator, the normalized permeability in Figure 8D.^18,23−26,33,44,48−61^ The antifouling property and the filtration performance of the TFC-10 membrane are improved together by in situ surface chemical modification with zwitterionic copolymer brushes, which are among the best in the current literature. It further confirms the effectiveness of this strategy to break the trade-off between filtration performance and antifouling property of the TFC NF membranes.

**Figure 9:**
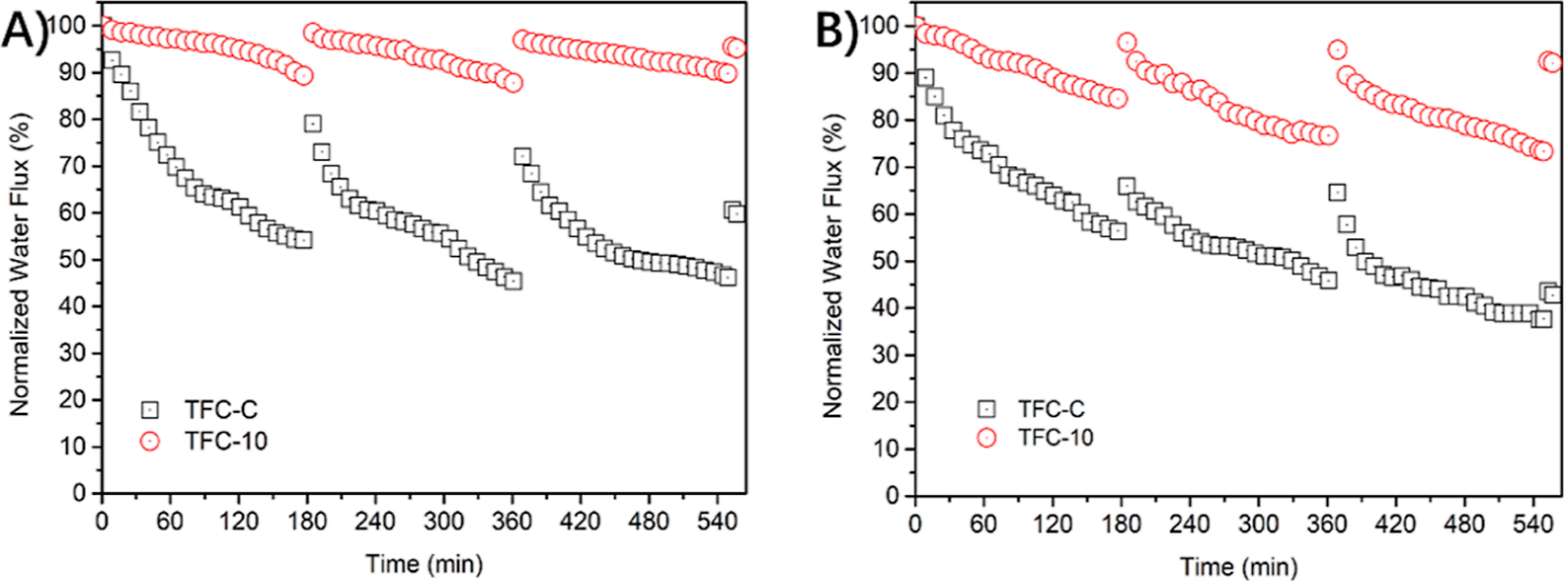
Water flux decline profiles for the TFC-10 membrane obtained from three cycles of (A) BSA and (B) HA fouling experiments. Fouling conditions were as follows: organic foulant (500 ppm) and inorganic composites (16 mM NaCl, 1 mM NaHCO_3_, and 1 mM CaCl_2_) were used as the feed solution. The initial water flux was fixed at 18 L m^–2^ h^–1^ by adjusting the operation pressure.

To further demonstrate the antifouling efficiency of the optimized NF membrane, three cycles of fouling tests for the TFC-10 membrane are carried out with BSA and HA. As expected, in both the BSA fouling tests (Figure 9A) and HA fouling tests (Figure 9B), the TFC-10 membrane shows both a much milder water flux decline and less irreversible membrane fouling than that of the pristine TFC NF membrane. This observation further validates the exceptional fouling resistance of zwitterionic copolymers on the polyamide surface. In addition, as for the TFC-10 membrane, HA fouling results are in a more severe flux decline than that of BSA fouling. The “Ca^2+^ ion bridging” effect can occur not only between the membrane surface and foulants but also between the foulants.^46,47^ This could be attributed to the difference between BSA and HA fouling behavior, where the HA aggregates and forms a more cross-linked HA on the polyamide surface. Despite such severe fouling potency, the TFC-10 membrane remains at 73% of its initial water flux after three HA fouling cycles and recovers to more than 92% after a simple physical cleaning process (see Figure S6), confirming the excellent antifouling properties of the zwitterionic copolymer-modified NF membrane.

## 4 Conclusions

In this work, we have demonstrated the successful fabrication of high-flux and antifouling TFC NF membranes by synthesizing a water-soluble zwitterionic copolymer with primary amine groups and chemically binding them to the nascent polyamide surface via the in situ surface chemical modification process. FT-IR and XPS analyses verified the successful grating of the zwitterionic copolymer. The surface wettability was enhanced first and then decreased with the increase in grafting concentration (the optimal grafting concentration was 1.0 wt %), while the membrane roughness increased gradually. The water permeability of the optimal modified membrane (i.e., TFC-10) came up to 27.5 L·m^–2^·h^–1^·bar^–1^, which was almost three times that of the pristine TFC membrane, while maintaining comparable Na_2_SO_4_ rejection. The increase in water permeability was attributed to the inhibition of zwitterionic copolymer grafting solution on the formation of an exterior layer that could impart the membrane with a larger actual surface area (i.e., nodular structures) and a thinner polyamide layer to promote the passage of water molecules. Furthermore, compared with the pristine TFC membrane, the TFC-10 membrane exhibited much lower foulant deposition and higher fouling resistance (i.e., a 66% decline in BSA adsorption), benefiting from the enhanced surface hydrophilicity after the grafting of the zwitterionic copolymer. After three cycles of BSA and HA fouling filtration tests, the TFC-10 membrane was still merely subjected to a milder flux decline and, more importantly, higher water flux resilience in comparison to the pristine TFC membrane. To recap, this pioneering work offers a versatile method for the design and fabrication of TFC NF membranes with simultaneously enhanced filtration performance and antifouling property.

## Supporting Information Available

Synthesis of the zwitterionic copolymer; gel permeation chromatography experiments; and surface characterization of AFM roughness (PDF)
