# Supplementary material for: In Situ Chemical Modification with Zwitterionic Copolymers of Nanofiltration Membranes: Cure for the Trade-Off between Filtration and Antifouling Performance
Source: ACS Appl Mater Interfaces. 2022 Jun 16;14(25):28842–53. doi: 10.1021/acsami.2c05311 (PMC9247986; doi:10.1021/acsami.2c05311)
Supplement: Supplementary file 1 — am2c05311_si_001.pdf [file am2c05311_si_001.pdf]

Supporting Information

In-situ Chemical Modification with Zwitterionic Copolymers of Nanofiltration Membrane: Cure for Trade-off between Filtration and Antifouling Performance

Xinyu Zhang[a], Jiayu Tian[b], Ruiyang Xu[c], Xiaoxiang Cheng[a], Xuewu Zhu[a], Ching Yoong Loh[d], Kaifang Fu[a], Ruidong Zhang[a], Daoji Wu[a,*] Huixue Ren[a,*], Ming Xie[d,*]

[a]*School of Civil and Environmental Engineering, Shandong Jianzhu University, 250101, PR China.*

[b]*School of Civil Engineering and Transportation, Hebei University of Technology, Tianjin 300401, PR China*

[c]*International Education School, Shandong Polytechnic College (SDPC), Jining 272100, PR China*

[d]*Department of Chemical Engineering, University of Bath, Bath BA27AY, UK*

*Corresponding authors. Tel.: +44(0)1225 383246. Email addresses: wdj@sdjzu.edu.cn (for Daoji Wu), renhx138@163.com (for Huixue Ren), mx406@bath.ac.uk (for Ming Xie).

Content

S1. Synthesis of the Zwitterionic Copolymer.

S2. Gel permeation chromatography experiments

S3 Surface characterization of AFM roughness

Figures

Figure S1. Schematic synthesis procedures for the zwitterionic copolymer

Figure S2. Water CA analyses performed after the nanofiltration membrane modified by zwitterionic copolymer with the grafting concentration of 1.0 wt% were subjected to stress.

Figure S3. FT-IR analyses performed after the TFC-10 membrane was subjected to stress.

Figure S4. Membrane performance performed after the TFC-10 membrane was subjected to stress.

Figure S5. Surface tension and viscosity of the zwitterionic copolymer solution with the different grafting concentration of 0 wt%, 0.5 wt%, 1.0 wt%, and 1.5 wt%.

Figure S6. Membrane fouling tests: FDR and FRR of (A) TFC membrane and (B) TFC-10 membrane by using BSA as foulant; FDR and FRR of (C) TFC membrane and (D) TFC-10 membrane by using HA as foulant.

S1 Synthesis of the Zwitterionic Copolymer

The zwitterionic random copolymer P[SBMA-co-AMA] was synthesized via a modified free radical polymerization between SBMA and AMA monomers using AIBN as the initiator [1]. In a typical reaction, SBMA (45 mmol,12.698 g), AEMA (5 mmol, 0.845 g) and AIBN (0.25 mmol, 0.0415g) were charged into a three-neck round-bottomed flask containing a water and DMSO mixture (2/1 by volume) of 300 ml. The mixture was then stirred and reacted at 70° C for 24 h under nitrogen protection. After the copolymerization, the reaction mixture was directly dialyzed (using a dialysis tubing cellulose membrane of MWCO 2000 Da) for 7 days with the deionized water changed twice daily. Finally, the purified mixture was freeze dried to obtain white powders of the P[SBMA-co-AEMA] copolymer.

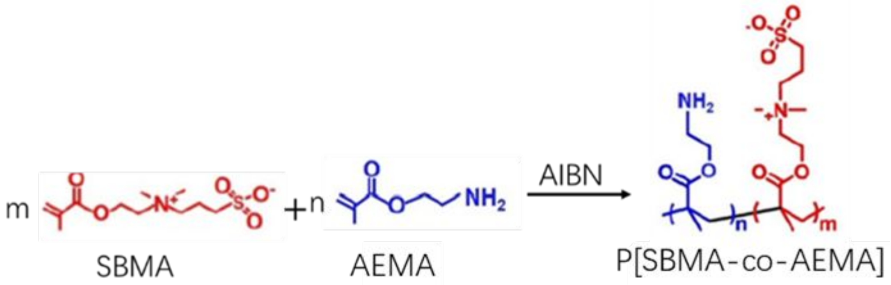

Figure S1 Schematic synthesis procedures for the zwitterionic copolymer

Reference

[1] G. Han, J. T. Liu, K. J. Lu, T. S. Chung, Advanced anti-fouling membranes for osmotic power generation from wastewater via pressure retarded osmosis (PRO), Environ. Sci. Technol. 52 (2018) 6686-6694.

S2 Gel permeation chromatography experiments

Gel permeation chromatography (GPC) experiments were carried out on a liquid chromatograph equipped with a Waters 2414 refractive-index detector and a Viscotek 270 RALLS/viscometric dual detector. DI Water was employed as the mobile phase at room temperature. Both the mobile phase and sample solution (zwitterionic copolymer) were filtered before introduction to the GPC system. Polymethyl mthacrylate was selected as the standards.

S3 Surface characterization of AFM roughness.

The membrane surface morphology was characterized by a multimode atomic force microscope (AFM, Dimension Edge, Bruker). Imaging of the air-dried samples was performed in tapping mode with silicon probes coated with 30-nm thick back side aluminum (Tap300A, Bruker Nano, Inc., Camarillo, CA). The probe had a spring constant of 40 N/m, resonance frequency of 300 kHz, tip radius of 8 ± 4 nm, and cantilever length of 125 ± 10 μm.

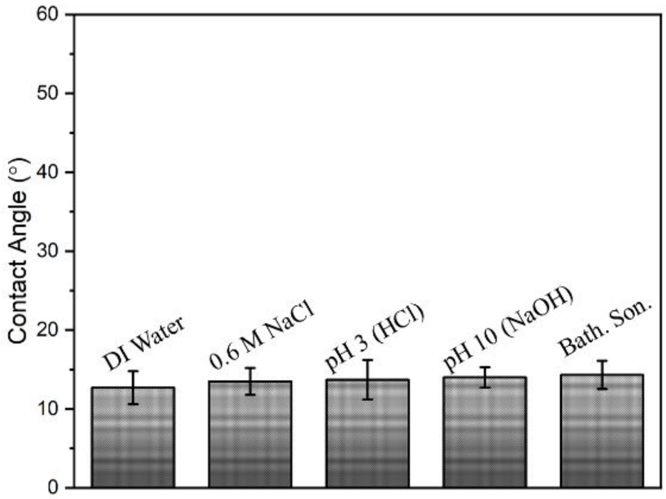

Figure S2 Water CA analyses performed after the TFC-10 membrane were subjected to stress.

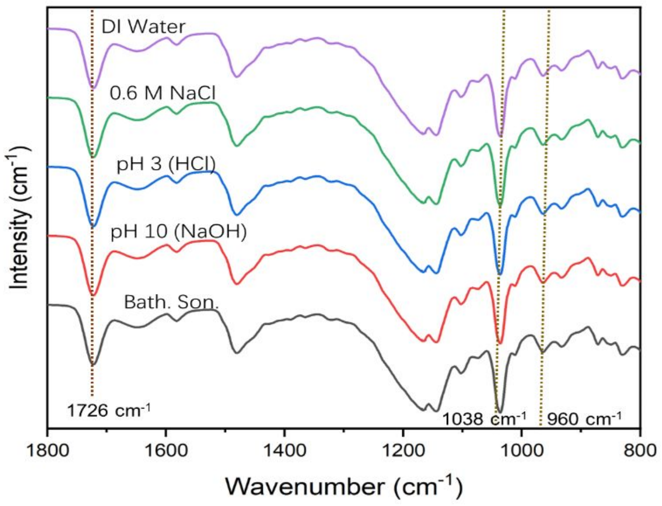

Figure S3 FT-IR analyses performed after the TFC-10 membrane was subjected to stress.

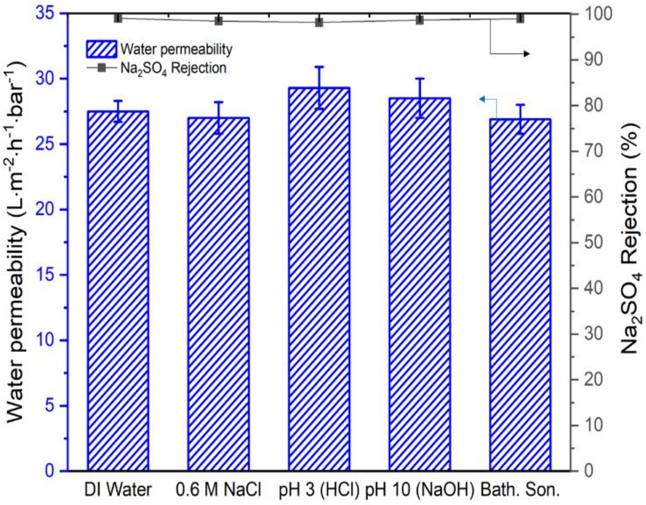

Figure S4 Membrane performance performed after the TFC-10 membrane was subjected to stress.

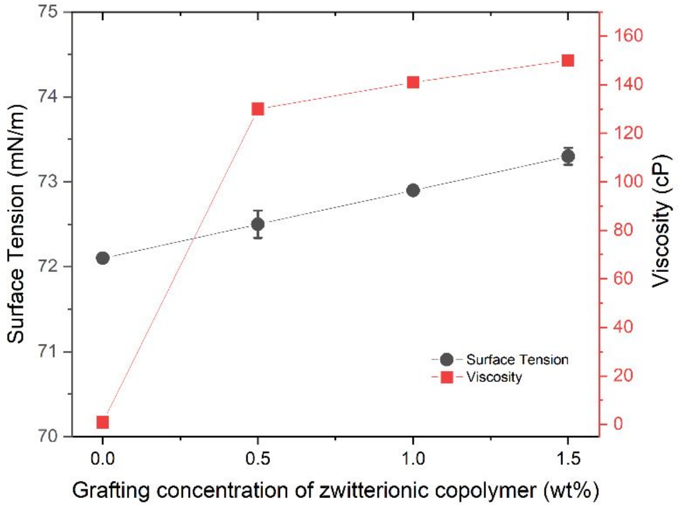

Figure S5 Surface tension and viscosity of the zwitterionic copolymer solution with the different grafting concentration of 0 wt%, 0.5 wt%, 1.0 wt%, and 1.5 wt%.

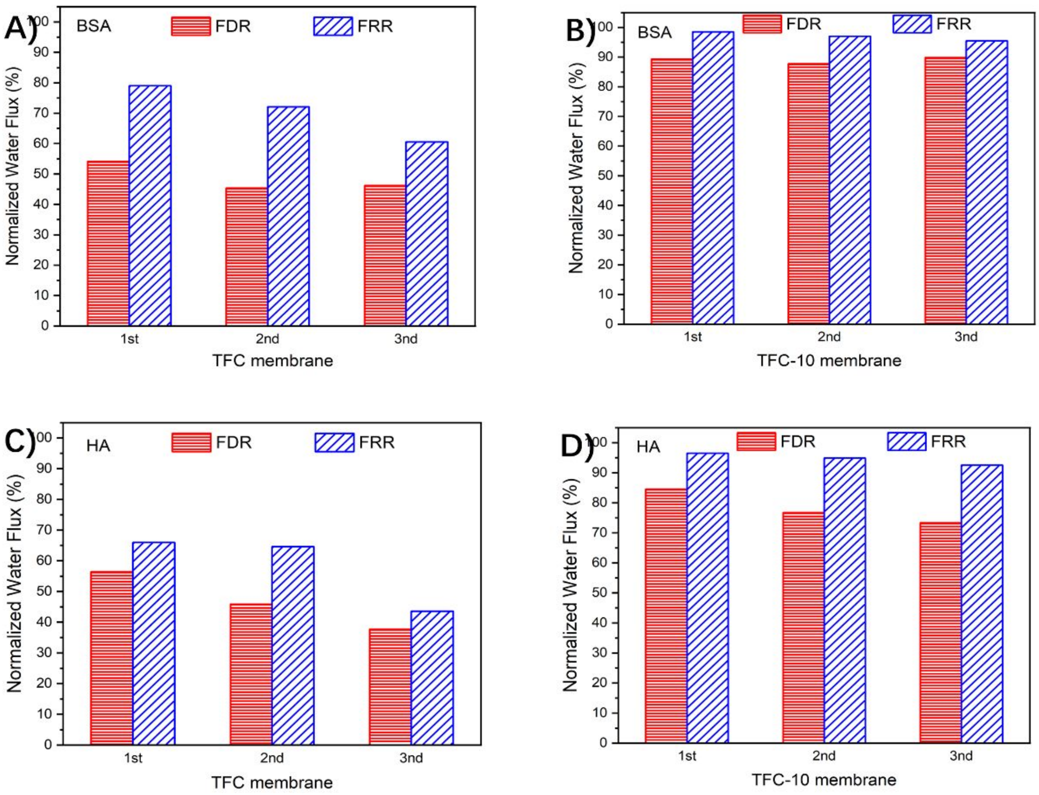

Figure S6 Membrane fouling tests: FDR and FRR of (A) TFC membrane and (B) TFC-10 membrane by using BSA as foulant; FDR and FRR of (C) TFC membrane and (D) TFC-10 membrane by using HA as foulant.
